# Gestational weight gain and its associated factors in Harari Regional State: Institution based cross-sectional study, Eastern Ethiopia

**DOI:** 10.1186/s12978-016-0225-x

**Published:** 2016-08-30

**Authors:** Fekede Asefa, Dereje Nemomsa

**Affiliations:** 1 Department of public Health, College of Health and Medical Sciences, Haranmaya University, Dire dawa, Ethiopia; 2 Federal Police Hospital, Harar, Ethiopia

**Keywords:** Gestational weight gain, Early pregnancy BMI, Recommended GWG

## Abstract

**Background:**

Gestational weight gain is an important factor that supports optimal outcome for mothers and their infant. Whereas women who do not gain enough weight during pregnancy have a risk of bearing a baby with low birth weight, those who gain excessive weight are at increased risk of preeclampsia and gestational diabetes. Nonetheless, data on gestational weight gain and its determinants are scarce in developing countries, as it is difficult to collect the information throughout the pregnancy period. Therefore, the aim of the study was to assess weight gain during pregnancy and its associated factors.

**Methods:**

The study employed a health facility based quantitative cross-sectional study design in Harari Regional State. The study included 411 women who had given birth at health institutions from January to July of 2014. The researchers collected both primary and secondary data by using a structured questionnaire and a checklist. Using logistic regression, the factors associated with gestational weight gain were assessed and, based on the United States Institute of Medicine criteria, gestational weight gains were categorized as inadequate, adequate and excessive.

**Results:**

The study revealed that 69.3 %, 28 %, and 2.7 % of the women gained inadequate, adequate and excess gestational weight, respectively. The mean gestational weight gain was 8.96 (SD ±3.27) kg. The factors associated with adequate gestational weight gain were body mass index ≥ 25Kg/m^2^ at early pregnancy (AOR = 3.2, 95 % CI 1.6, 6.3); engaging in regular physical exercise (AOR = 2.1, 95 % CI 1.2, 3.6); Antenatal care visit of ≥4 times (AOR = 2.9, 95 % CI 1.7, 5.2); consuming fruit and vegetable (AOR = 2.7, 95 % CI 1.2, 6.6), and meat (AOR = 2.7, 95 % CI 1.1, 97.2).

**Conclusions:**

Generally, a small proportion of the women gained adequate gestational weight. The women who were with higher body mass index at early pregnancy, who frequently visited Antenatal care visit, and who consumed diverse food items were more likely to measure adequate gestational weight.

## Plain english summary

Gestational weight gain is an important factor that is  required to support increased metabolic demands, and to enhance positive pregnancy outcomes. A desirable gestational weight gain is essential for a balanced optimal outcome for both the mother and her infant. In contrast, inadequate or excessive weight gain may pose health risks on the mother and/or the fetus.

The study included 411 women who had given birth at health institutions from January to July of 2014. The researchers collected both primary and secondary data by using a structured questionnaire and a checklist. Based on the United States Institute of Medicine criteria, gestational weight gains were categorized as inadequate, adequate and excessive.

The study revealed that 69.3 %, 28 %, and 2.7 % of the women gained inadequate, adequate and excess gestational weight, respectively. The average gestational weight gain was 8.96 kg. The factors associated with adequate gestational weight gain were high body mass index at early pregnancy; engaging in regular physical activity; Antenatal care visit of ≥4 times; consuming fruit and vegetable, and meat.

Generally, a small proportion of the women gained adequate gestational weight. The women who were with higher body mass index at early pregnancy, who frequently visited Antenatal care visit, and who consumed diverse food items were more likely to measure adequate gestational weight.

## Background

Gestational weight gain (GWG) is an important factor that is required to support increased metabolic demands, and to enhance positive pregnancy outcomes [1]. A desirable GWG is essential for a balanced optimal outcome for both the mother and her infant [2]. It supports the growth and development of the fetus [3], and reduces the likelihood of morbidity and mortality [2, 4]. In contrast, inadequate or excessive weight gain may pose health risks on the mother and/or the fetus [5].

GWG is highly influenced by a range of biological, metabolic, and social factors, which include maternal pre-pregnancy body mass index (BMI) [6], multi-parity [6, 7], maternal age, smoking, educational status [3], healthy eating, physical activity [2], and adequate counseling of mothers on weight gain during pregnancy [8]. However, in developing countries, there is very little information about GWG and its determinants. This is most likely due to the difficulties associated with collecting the data throughout the pregnancy period in these settings [9], and as such GWG remains a neglected public health issue in the developing world. Therefore, the aim of this study was to assess GWG and its associated factors in the health care facilities in Harari Regional State, Eastern Ethiopia.

## Methods

### Study setting

The study was conducted in Harari Regional State, whose capital, Harar, lies 526 km to the East of Addis Ababa. According to 2015 Ethiopian Central Statistical Agency population projection, the region has a population of 232,000, of whom 127,600 (55 %) are urban dwellers. According to the Regional Health Bureau, the health service coverage of the region is 100 %. There are two public hospitals, one Federal Police Hospital, one Federal Defense Hospital, two private General hospitals, one Fistula Hospital, eight government health centers, 16 health posts, and one nongovernmental organization clinic. The health worker per 1,000-population ratio is 2.8.

### Subjects

We employed a health facility based quantitative cross-sectional study from January to July of 2014. The study included Hiwot Fana Hospital, Jugal Hospital, Harar Federal Police Hospital, Family Guidance Association Harar Model Clinic, and Arategna Health Centre. The pregnant women who attended antenatal care (ANC) clinic during first trimester (started their ANC visit at ≤16 weeks of gestation) and who gave live birth in the health care facilities were included in the study. Women with twin pregnancy were excluded. The target sample size was determined by Open Epi Version 2.3, by taking the proportion of the women who gained adequate gestational weight (*p* = 0.55) [10], 5 % margin of error, 95 % confidence level, and 10 % non-response rate. The final sample size was 418. All the women who gave birth and fulfilled the inclusion criteria were included in the study until the required sample size was achieved.

### Measurements

We collected both the primary and the secondary data through interview and checklists. Secondary data like initial maternal weight, gestational age, number of ANC visit, and parity were extracted from antenatal follow up registration card. The last maternal weight (just before delivery) was measured by digital weight scale with minimum clothing. A participants’ first measured weight (before or at 16 weeks’ gestation) was used as the proxy for her weight at conception. GWG is the difference between the last and the first measured maternal weight. The United States Institute of Medicine (IOM) classifies a healthy GWG of 12.5–18 kg, 11.5–16 kg, 7–11 kg and 5–9 kg for underweight, normal, overweight and obese women respectively. Accordingly, weight gains below and above these recommendations are considered inadequate and excessive [3]. Variables, which collected through interviewing, were socio-demographic and economic characteristics, dietary pattern/dietary habit and physical exercise. Women were asked for their average dietary habit and physical activity throughout pregnancy.

### Data quality control

The questionnaire was pre-tested on 20 women in Dilchora Hospital at Dire Dawa (outside of the study area) and feedback was used to guide the modifications necessary to optimise the questionnaire. The data collectors (midwives) and the supervisors (health officers) were selected from the health facilities based on their qualifications and field data collection experience. They were given training on the objectives of the study, data collection methods, and field supervision. The supervisors and principal investigator checked data for completeness on daily basis.

### Data processing and analyses

Data were entered into Epi-data Version 3.0 and analysed using SPSS 20 statistical packages. Frequencies, proportions, measures of central tendency, and dispersions were estimated to describe the variables. Crude odds ratios (COR) and adjusted odds ratios (AOR) were calculated to determine the association between the explanatory variables and GWG. The variables associated with the dependent variable in the bivariate analyses at p ≤0.2 were entered into multivariable logistic regression model. The total GWG was taken as the difference between the last measured weight (recorded just before delivery) and the first measured weight at early pregnancy (before or at first trimester). Based on the IOM criteria, GWG was categorised as inadequate, adequate and excessive. Eleven (2.7 %) respondents who gained excessive weight were excluded from the logistic regression analyses.

## Results

### Socio-demographic characteristics

Out of the 418 participants identified for the study, 411 were included in the study, which gives a response rate of 98.3 %. Their mean age was 25.2 (SD ±5.01) years, and 65.2 % of them were between 20 and 29 years of age. Most of the respondents (94.6 %) were married, 25.8 % were illiterate, 16.5 % attended tertiary education, 44 % were Oromo in ethnic, 55.2 % were Muslim, 81 % were urban residents, and 52.6 % were homemakers. Eighty four percent of the respondents started ANC follow up between 8 and 12 weeks of gestational age (Table 1).

**Table 1:** Socio-demographic characteristics of the respondents in Harari Regional state, 2014 (*n* = 411)

Variable	Frequency	percent
Age group (year)		
less than 20	49	11.9
20–29	268	65.2
> 29	94	22.9
Marital status		
Single	18	4.4
Married	389	94.6
Divorced	2	0.5
Widowed	2	0.5
Educational status		
No formal education	106	25.8
Primary and secondary	237	57.7
Tertiary education	68	16.5
Ethnicity		
Amhara	128	31.1
Oromo	181	44
Gurage	42	10.2
Harari	35	8.5
Tigrai	15	3.6
Others ^a^	10	2.4
Religion		
Muslim	227	55.2
Christian	181	44
Others	3	0.7
Residence		
Rural	78	19
Urban	333	81
Occupational status		
Homemaker	216	52.6
Government employee	80	19.5
Merchant	49	11.9
Private employee	28	6.8
Farmer	23	5.6
Student	7	1.7
Daily laborer	8	1.9
Estimated income (USD)		
Less than $50	88	21.5
$50–$100	157	38.2
> $100	166	40.3
Gestation age at ANC initiation		
Less than 8 weeks	39	9.5
8–12 weeks	345	83.9
13–16 weeks	27	6.6

^a^ Others - Somali, Walayita, Argoba

### Eating habit and physical exercise of the respondents

Only 16.5 % of the women ate food at least three times a day during their current pregnancy, and 81.5 %, 79.1 %, and 91.7 % of them consumed fruits and vegetables, meat, and egg at least once a week, respectively. During their current pregnancy, 65.2 % were not engaged in any physical activities (Table 2).

**Table 2:** Eating habit and physical exercise  of the respondents during their current pregnancy in Harari Regional State, 2014 (*n* = 411)

Variable	Frequency	Percept
Frequency of eating per day		
< 3 times	343	83.5
≥ 3 times	68	16.5
Frequency of eating vegetables and fruit at least once per week		
Yes	335	81.5
No	76	18.5
Frequency of eating meat at least once per week		
Yes	325	79.1
No	86	20.9
Frequency of eating egg at least once per week		
Yes	377	91.7
No	34	8.3
Physical exercise at least once per week		
Yes	143	34.8
No	268	65.2

### Early pregnancy BMI and GWG of the study participants

The mean BMI of the respondents at early pregnancy was 22.39 (SD ± 3.84 kg/m^2^), and 72 % of them had a normal body weight (BMI 18–24.9 kg/m2), whereas 14.6 % were overweight (BMI 25–29.9 kg/m2). The mean weight gain during their pregnancy was 8.96 (SD ±3.27 kg) kg. Underweight and obese women gained 9.14 (SD ±3.46 kg) and 6.44 (SD ±3.46 kg), respectively (Table 3). Many of the women (69.3 %) gained inadequate gestational weight, but only 11 (2.7 %) respondents gained excessive gestational weight. Based on early pregnancy BMI, only 7.7 % of the underweight women, 24 % of the women with normal BMI, 51.7 % of the overweight women, and 62.5 % of the obese women gained adequate gestational weight (Table 4).

**Table 3:** BMI at early pregnancy and mean gestational weight gain in Harari Regional state, 2014 (*n* = 411)

Early pregnancy BMI	Frequency (%)	Mean GWG	SD
<18.5 kg/m^2^	39 (9.5)	9.14 kg	±3.46 kg
18.5–24.9 kg/m^2^	296 (72)	9.26 kg	±3.14 kg
25–29.9 kg/m^2^	60 (14.6)	8.03 kg	±3. 64 kg
≥30/m^2^	16 (3.9)	6.44 kg	±3.46 kg
Total	411 (100)	8.96 kg	±3.27 kg

**Table 4:** Proportion of gestational weight gain of the women based on early pregnancy BMI in Harari Regional state, 2014 (*n* = 411)

Early Pregnancy BMI	Inadequate GWG	Adequate GWG	Excess GWG
	*N* (%)	*N* (%)	*N* (%)
Under weight	35 (89.7)	3 (7.7)	1 (2.6)
Normal	222 (75)	71 (24)	3 (1)
Overweight	23 (38.3)	31 (51.7)	6 (10)
Obese	5 (3.25)	10 (62.5)	1 (6.25)
Total	285 (69.3)	115 (28)	11 (2.7)

### Factors associated with weight gain during pregnancy

In a logistic regression model, the women who had higher early pregnancy BMI (overweight and obese) were more likely to gain adequate gestational weight as compared to the underweight mothers (AOR = 3.2,95 % CI 1.6,6.3). The women who fed on fruit and vegetables (AOR =2.7, 95 % CI 1.16, 6.6), or meat (AOR = 2.7,95 % CI 1.1,7.2) at least once a week were more likely to gain adequate gestational weight than their counter parts, as were women who engaged in different physical activities (AOR = 2.1, 95 % CI 1.2, 3.6). Likewise, women who gave birth at or after 37 weeks (AOR = 4.5, 95 % CI1.1, 20.7), or who had ANC follow up of ≥4 times (AOR = 2.9, 95 % CI 1.7, 5.1) were more likely to gain adequate gestational weight compared to their counterparts (Table 5).

**Table 5:** Factors associated with gestational weight gain during pregnancy of Harari Regional State, 2014 (*N* = 400)

Variable	Adequate GWG	Inadequate GWG	COR (95 %,CI)	AOR (95 % CI)
Early pregnancy BMI				
Underweight	3 (7.9)	35 (92.3)	1.00	1.00
Normal	71 (24.2)	222 (75.8)	17 (4.7–61)*	7.9 (1.9–34)***
Overweight& obese	41 (59.4)	28 (41.6)	4.6 (2.6–7.9)***	3.2 (1.6–6.3)*
Gestational age				
<37 weeks	*2 (3.7)*	52 (96.3)	1.00	1.00
≥37 weeks	*113 (32.7)*	233 (67.3)	12.6 (3–52)**	4.5 (1.1–20.7)*
ANC visit				
≤3	*29 (14.9)*	166 (85.1)	1.00	1.00
≥4	*86 (42)*	119 (58)	4 (2.5–6.7)***	2.9 (1.7–5.2)***
Physical exercise per week				
Not at all	*57 (21.9)*	*203 (78.1)*	1.00	1.00
At least once	*58 (41.4)*	*82 (58.6)*	2.5 (1.6–3.9)***	2.1 (1.2–3.6)*
Monthly Income status				
< $50	*17 (19.3)*	71 (80.7)	1.00	1.00
$50–$100	*17 (11.2)*	135 (88.8)	0.5 (0.3–1.1)	1.5 (0.7–3.3)
> $100	*81 (50.6)*	79 (49.4)	2.7 (1.4–5.2)**	5.2 (2.7–9.9)
Frequency of eating a day				
<3times/day	*85 (25.2)*	252 (74.8)	1.00	1.00
≥3 times/day	*30 (47.6)*	33 (52.4)	2.6 (1.6,4–68)**	2.5 (0.9–2.4)
Fruit and vegetables consumption per week				
Not at all	*9 (12.2)*	65 (87.8)	1.00	1.00
At least once	*106 (32.5)*	220 (67.5)	3.5 (1.7–7.3)**	2.7 (1.2–6.6)*
Eggs consumption per week				
Not at all	*5 (15.2)*	28 (84.8)	1.00	1.00
At least once	*110 (30)*	257 (70)	2.4 (0.9,6.37)	0.4 (0.1,1.5)
Meat consumption per week				
Not at all	*9 (10.7)*	75 (89.3)	1.00	1.00
At least once	*106 (33.5)*	210 (66.5)	4 (2.0–8.7)***	2.7 (1.1–7.2)*
Residence				
Rural	*15 (21.4)*	55 (78.6)	1.00	1.00
Urban	*100 (30.3)*	230 (69.7)	1.6 (0.9–2.9)	0.8 (0.4–1.8)
Parity				
primipara	54 (25.5)	158 (74.5)	1.00	1.00
Multi para	61 (32.4)	127 (67.6)	1.4 (0.9–2.2)	0.9 (0.5–1.7)

*,*p*=,0.05,** *p* <0.01.*** *p* <0.001

## Discussion

Adequate gestational weight gain is required for optimal pregnancy outcome. Less than one-third (28 %) of the pregnant women in this study gained adequate gestational weight. The factors positively associated with the adequate gain of the weight were having high BMI at early pregnancy (≥25 kg/m^2^), engaging in regular physical exercise, visiting ANC frequently, and eating fruit, vegetables and meat.

In the study, the mean weight gain during pregnancy was 8.96 kg. This is consistent with the findings from a study in Pakistan (8.5 kg) [11], but less than the findings from research in Brazil, in which the mean GWG ranged from 11.7 to 13.9 kg depending on BMI at early pregnancy [12–14]. Although the proportion of the women who gained inadequate (69.3 %) and adequate (28 %) gestational weight in our study is similar to those found in studies conducted in other countries [15, 16], it is smaller than the findings of similar studies conducted in other areas [11, 14, 17, 18]. The inadequate gestational weight gain in our study is most likely related to the nutritional condition of the women, given that 84 % of the women had less than three meals per day. In addition, a significant proportion of women were underweight at the conception. Moreover, while IOM recommendations may be well suited to high-income countries, there may be a number of contextual factors that limit the applicability of these guidelines to low or middle income-countries such as Ethiopia. It is, therefore, important to consider GWG in the context of all the factors in a woman’s life and to develop a GWG guideline that could address the context of developing countries.

This study also indicated that the women with higher early pregnancy BMI were more likely to gain adequate gestational weight when compared to underweight women. This is consistent with previous research in Pakistan [11]. Women who are underweight at early pregnancy are required to gain more weight than their overweight or obese counterparts do in order to achieve a healthy GWG. It may be difficult for these women to gain a significant amount of weight during pregnancy, particularly if they tend to be underweight due to metabolic or food security factors. Overweight and obese women, on the other hand, are required to gain comparatively little weight to achieve adequate GWG as they are able to use a portion of their stored energy to support the growth of the fetus. As such, adequate GWG may be attained easily for these women.

At least a half-an- hour of physical exercise per day is believed to be vital during pregnancy for a healthy lifestyle and for weight management for both the mother and the fetus [19]. In the study, the mothers who undertook physical exercise at least once a week were 2.1 times more likely to gain adequate gestational weight compared to those that did not. This finding is in line with other studies conducted in the United states [20] and China [21].

The women who frequently visited ANC (≥4 times) were 2.9 times more likely to gain adequate gestational weight. This result supports the findings of the study in southern Brazil [15]. This may be explained by the fact that during ANC visits, women are likely to receive advice on weight management, the importance of maintaining a balanced diet, the need for proper nutrition during pregnancy. The women who gave birth at or after 37 weeks of gestation were 4.5 times more likely to have gained adequate gestational weight compared to than those who gave birth before 37 weeks. This is supported by research in Thailand [6], and may be attributed to an increased opportunity to gain weight by virtue of having an increase gestational period.

Women who eat fruit and vegetable for at least once per week were 2.7 times more likely to gain adequate gestational weight. This contrasts with finding of another study [20] in which fruit and vegetable consumption during pregnancy has no association with weight gain. The difference might be due to a small number of subjects (105) in the indicated study. However, in this study, 81.5 % of the women consumed fruit and vegetable most of the day during their current pregnancy.

The women from good family income (family income of > $100 per month) were 5 times more likely to gain adequate gestational weight than their counter parts, a finding supported by research in Southern Brazil [15]. It is likely that a good family income enhances household food security. In which case, pregnant women with a high family income are more likely to have consistent access to a varied diet and thus could gain appropriate weight during pregnancy.

This study has some limitations. Since the weight gain recommendation is the recommendation of developed countries, it may underestimate the proportion of gestational weight gain in developing countries such as Ethiopia, where this study was conducted. Moreover, early pregnancy BMI was taken before or at 16 weeks of gestation, at which time there may already have been an increase or decrease of gestational weight. Since variables like physical excersise and dietary habits were asked retrospectively, and they are qualitative in nature; it is difficult to measure objectively and does not indicate any specific trimester. Findings are also generalizable only for women who attended at least one ANC visit and gave birth at a health facility.

## Conclusions

A considerable proportion of women in this study (69.3 %) gained inadequate gestational weight, while less than one-third (28 %) gained adequate gestational weight. Women with higher BMI at early pregnancy, who frequently visited ANC, or who consumed diverse food items during pregnancy have higher likelihood of gaining recommended gestational weight. Therefore, women of childbearing age should be informed about the importance of conceiving at a normal BMI, maintaining a balanced diet, engaging in physical activity during pregnancy, and achieving a healthy gestational weight gain. There is also a need to develop guidelines on gestational weight gain to optimize pregnancy and birth outcomes in low- and middle-income countries.

## Abbreviations

ANC: Antenatal care
AOR: Adjusted odds ratio
BMI: Body mass index
CI: Confidence interval
COR: Crude odds ratio
GWG: Gestational weight gain
IOM: Institute of medicine
IRERC: Institutional Research and Ethical Review Committee
SD: Standard deviation
SPSS: Statistical package for social science
